# Exoskeleton for Upper Limb Rehabilitation (EULR) with 3D printing technology based on force sensor

**DOI:** 10.1016/j.ohx.2025.e00665

**Published:** 2025-06-14

**Authors:** Triwiyanto Triwiyanto, Levana Forra Wakidi, I. Putu Alit Pawana

**Affiliations:** a Department of Medical Electronics Technology, Poltekkes Kemenkes Surabaya, Indonesia; b Intelligent Medical Rehabilitation Devices Research Group, Department of Medical Electronics Technology, Poltekkes Kemenkes Surabaya, Indonesia; c Faculty of Medicine, Universitas Airlangga, Surabaya, Indonesia

**Keywords:** Exoskeleton, Bilateral rehabilitation, Force sensor, Post-stroke, *Microcontroller*

## Abstract

The paper addresses the significant challenge of limited accessibility and high costs associated with commercial exoskeletons for hand rehabilitation, particularly for individuals with low to middle incomes. The aim of this study is to design and develop a low-cost, 3D-printed hand exoskeleton that integrates force sensor technology, providing a more adaptable solution for rehabilitation. The methodology involves creating a prototype that combines 3D printing with real-time monitoring of upper limb (elbow) movements and forces, ensuring personalized treatment for patients. The design incorporates a lightweight structure, powered by a rechargeable LiPo battery, and utilizes mini ESP32 microcontrollers to collect the sensor parameters and drive the servo motor, enhancing user experience and functionality. Results indicate that the proposed exoskeleton significantly reduces costs to approximately 98.4 US$ per unit, compared to existing products priced above 1,500 USD. The mean root mean square error (RMSE) for the exoskeleton’s finger movements was measured at 0.498° ± 0.709°, demonstrating high accuracy in tracking hand movements. The mean linearity error of load cell across all data points was 0.2292 %. These results indicate that the load cell maintains good linearity and accuracy within the calibrated range, and is suitable for precise force measurements in static applications. Additionally, the integration of force sensors allows for precise feedback during rehabilitation exercises, promoting better outcomes. The study concludes that this innovative approach not only makes hand rehabilitation more accessible but also encourages further research and development in the field. By providing an open-source design, the research fosters collaboration among researchers and developers, paving the way for future enhancements and adaptations of the exoskeleton to meet diverse patient needs. Overall, this work contributes to advancing rehabilitation technology, ultimately improving the quality of life for individuals recovering from neuromuscular disorders.

## Graphical abstract

**Figure:**
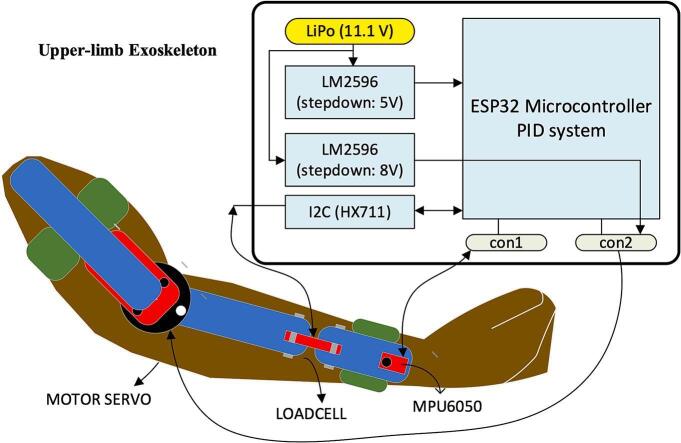

**Specifications table:** 

Hardware name	*Exoskeleton for Upper Limb Rehabilitation (EULR) with 3D Printing Technology Based on Force Sensor*
Subject area	• *Electronics and Microcontroller system* • *Rehabilitation Engineering*
Hardware type	• Measuring physical properties and in-lab sensors • Field measurements and sensors • Electrical engineering and computer science • Mechanical engineering and materials science • *Mechatronic engineering*
Closest commercial analog	*This hardware provide wireless control from another healthy hand*
Open source license	*https://creativecommons.org/licenses/by-sa/4.0/*
Cost of hardware	98.4 *US$*
Source file repository	DOI https://doi.org/10.17605/OSF.IO/H8GQK
OSHWA certification UID	ID000010

## 1 Hardware in context

The human hand is a complex and highly functional structure, essential for performing everyday activities such as grasping, holding, and manipulating objects. Unfortunately, conditions like stroke, spinal cord injuries, or other neuromuscular disorders can impair hand mobility, significantly reducing a patient's quality of life [[1], [2], [3], [4], [5], [6]]. Rehabilitation therapies are critical to helping patients regain their lost motor functions [1,[6], [7], [8]]. However, traditional hand rehabilitation methods, which often rely on human therapists and repetitive exercises, face limitations such as high costs, time constraints, and limited accessibility. With the rise of technology in the medical field, hand exoskeletons have emerged as a promising solution to enhance rehabilitation processes, offering more precise and controlled assistance to patients undergoing therapy. In recent years, state-of-the-art methods in hand rehabilitation have explored the integration of robotics and 3D printing technologies [[9], [10], [11], [12], [13], [14], [15]]. Exoskeletons equipped with sensors and actuators provide controlled force and movement, helping patients perform complex hand movements even when they are unable to do so independently. One of the most advanced methods in this domain is the use of force sensors in conjunction with 3D-printed components.

**Fig. 1:**
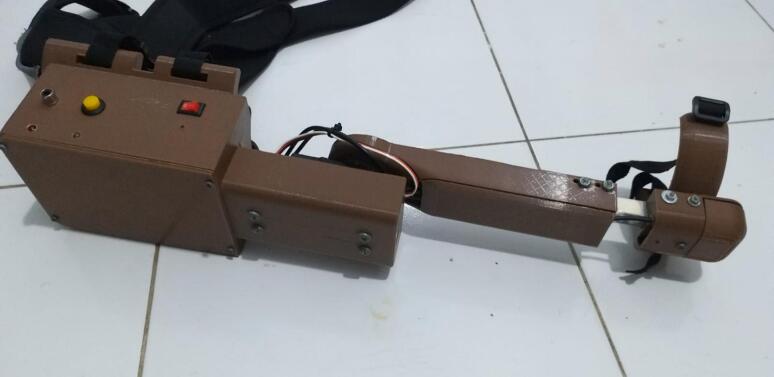
The proposed hardware of the Exoskeleton for Upper Limb Rehabilitation (elbow) (EULR) with 3D Printing Technology Based on Force Sensor.

Force sensors allow the exoskeleton to detect the amount of pressure exerted by the hand, which enables more accurate control over the rehabilitation process (Fig. 1). Additionally, 3D printing has revolutionized exoskeleton design, offering customizability, affordability, and faster prototyping for devices that fit patients' specific anatomical needs [[16], [17], [18]]. Despite the significant advancements in this field, there remain several research gaps that need to be addressed. Most current exoskeleton designs, while functional, are often expensive and not easily accessible for home-based rehabilitation. Moreover, they are typically designed for general hand rehabilitation and may not account for individual variations in hand structure or injury type. Another gap lies in the integration of force sensors; many existing designs either lack precise sensor-based feedback or focus solely on movement assistance without considering the force exerted by the patient. As a result, these designs may fail to provide the optimal level of assistance, potentially leading to ineffective rehabilitation outcomes.

The aim of this study is to design and develop a low-cost, 3D-printed hand exoskeleton that is based on force sensor technology to provide a more accessible and adaptable solution for hand rehabilitation. By combining 3D printing with force sensor integration, the proposed exoskeleton will enable precise monitoring of the user's hand movements and forces, allowing for better rehabilitation outcomes. Additionally, this study seeks to address the gaps identified in previous research by focusing on creating an exoskeleton that is both affordable and suitable for home use, making it more accessible to a wider population of patients in need of rehabilitation therapy. The key contribution of this research lies in the novel combination of force sensors and 3D-printed exoskeletons specifically designed for hand rehabilitation. This study will explore the design, development, and testing of a prototype that is adaptable to different hand sizes and conditions, ensuring personalized treatment for patients. Furthermore, by making use of 3D printing technology, this project will aim to reduce the cost and increase the accessibility of hand exoskeletons for rehabilitation. The integration of force sensors is expected to enhance the exoskeleton's ability to provide real-time feedback, improving the overall effectiveness of rehabilitation and promoting faster recovery. In sum, this research intends to advance the current state-of-the-art techniques and offer a more practical and cost-effective solution for individuals requiring hand rehabilitation.

## 2 Hardware description

**Fig. 2:**
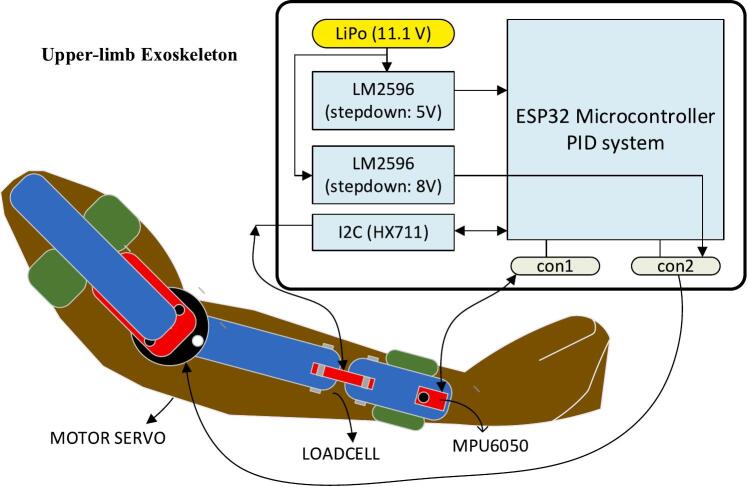
The diagram block of the Exoskeleton for Upper Limb (elbow) Rehabilitation (EULR) with 3D Printing Technology Based on Force Sensor.

The block diagram presented illustrates the design of an upper-limb exoskeleton based on 3D printing technology (Fig. 2). The primary components of this system include mechanical, electrical, and control subsystems that work together to support and enhance upper-limb rehabilitation. The exoskeleton is designed to be powered by a rechargeable LiPo battery (11.1 V), which provides the necessary energy for the various electronics and actuators within the system. The power from the LiPo battery is distributed to different components using two LM2596 step-down voltage regulators. One regulator steps the voltage down to 5 V, which is suitable for lower-power components such as sensors and the microcontroller.

The second regulator steps the voltage down to 8 V, which likely powers more power-intensive components such as the motor or servo actuators. These voltage regulators ensure that the individual modules receive the correct operating voltage, protecting the system from damage due to overvoltage. At the core of the exoskeleton’s control system is the ESP32 microcontroller, which manages the system’s logic, data processing, and communication. The ESP32 is responsible for executing a PID (Proportional-Integral-Derivative) control algorithm to maintain precise control over the motor servo and other dynamic elements of the exoskeleton. The PID system helps in achieving smooth and controlled movements, essential for rehabilitation tasks. It uses real-time data from sensors, such as a load cell and an MPU6050, to make necessary adjustments. The load cell measures the force or pressure applied by the arm, which is crucial for determining the effort the user is putting into the movement. The MPU6050 provides orientation and motion data by combining an accelerometer and gyroscope, allowing the system to understand the position and speed of the user’s arm. The diagram shows a motor servo connected to the upper-limb exoskeleton, which provides the necessary mechanical force to assist or resist the user's arm movements during rehabilitation exercises. The servo motor works under the control of the PID system, ensuring smooth and accurate motion control. This motor is essential for providing active support, such as assisting the user in lifting the arm or performing complex movements that might be difficult or impossible due to muscle weakness or injury. The exoskeleton employs two key sensors: the MPU6050 and a load cell. The MPU6050 sensor provides feedback on the arm's angular position, motion, and orientation, helping the system adjust movements in real-time for proper alignment and to prevent unintended movements. The load cell sensor measures the force exerted by the user during the movement. This force data is fed into the control system, allowing for adaptive resistance or assistance to be provided to the user based on their strength levels. The sensors ensure the exoskeleton responds dynamically to the user’s capabilities, which is particularly useful in rehabilitation applications where the user’s strength may vary day-to-day. The microcontroller communicates with the sensors via an I2C (Inter-Integrated Circuit) bus through an HX711 interface, which amplifies the signal from the load cell, making it readable for the microcontroller. The I2C bus is a crucial component as it allows multiple devices (sensors, motor drivers) to communicate with the microcontroller using only two data lines, reducing the complexity of wiring within the system. A significant advantage of this exoskeleton system is its use of 3D printing technology. The mechanical parts of the exoskeleton can be custom-designed and manufactured to fit the user’s anatomy, making the system highly adaptable and cost-effective. 3D printing allows rapid prototyping and customization, which is essential in rehabilitation devices where patient-specific adjustments are often required. This modularity not only reduces production costs but also allows for repairs and modifications to be easily carried out, enhancing the longevity and usability of the exoskeleton.

The diagram shows an upper-limb exoskeleton system controlled by an ESP32 microcontroller using a PID control system. The LiPo battery (11.1 V) provides power to the system, and two LM2596 voltage regulators step down the voltage to 5 V and 8 V, respectively, for different components. The 5 V output powers the ESP32 microcontroller and associated sensors. The ESP32 handles the PID control system, which regulates the movement of the exoskeleton based on feedback from sensors. The PID control parameters were determined through an empirical tuning process. Specifically, we employed a trial-and-error approach, adjusting the proportional, integral, and derivative gains iteratively based on system response tests to achieve smooth and stable control of the exoskeleton movements. This manual tuning process allowed us to tailor the control parameters to the specific dynamics of our prototype, ensuring responsive and safe operation suitable for rehabilitation purposes.

The I2C (HX711) module is used to read data from the load cell, which measures the force exerted by the exoskeleton. The MPU6050 sensor is used to detect motion, acceleration, and orientation of the arm, which is critical for precise control. The motor servo drives the exoskeleton’s movement by executing commands from the microcontroller, and the load cell ensures that the correct amount of force is applied, protecting the user. The connections labeled “con1” and “con2” refer to the wiring interfaces connecting the motor servo and sensors to the ESP32 microcontroller, completing the feedback loop needed for real-time adjustments in arm support and motion.

### 2.1 Exoskeleton design

**Fig. 3:**
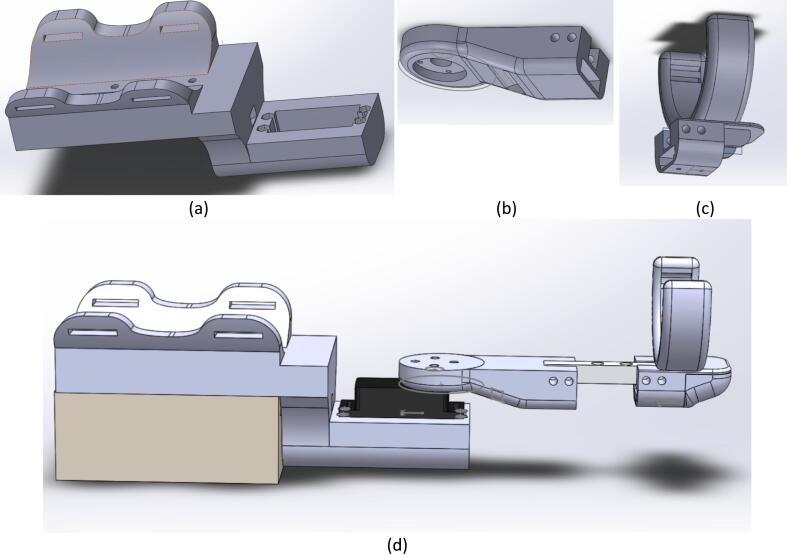
Design of Exoskeleton for Upper Limb Rehabilitation (EULR) with 3D Printing Technology Based on Force Sensor using Solidworks application.

**Fig. 4:**
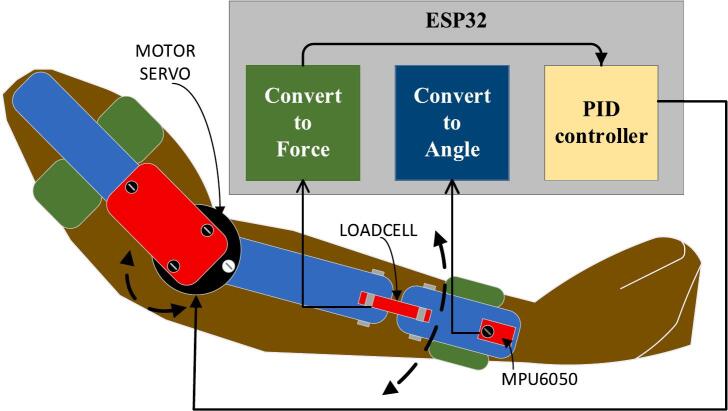
Exoskeleton for Upper Limb Rehabilitation (EULR) mechanical and circuit.

The Exoskeleton for Upper Limb (elbow) Rehabilitation (EULR) was developed using the 3D design software Solidworks (Solidworks Premium, SP05 64-bit, 2020 Dassault Systèmes SOLIDWORKS Corp, US and Canada), as depicted in Fig. 3. The design is comprised of three primary components: the circuit and motor holder (Fig. 3(a)), the motor link (Fig. 3(b)), and the wrist holder (Fig. 3(c)), with the complete model illustrated in Fig. 3(d). The circuit and motor holder securely accommodates the circuit box and motor servo (Fig. 3(a)). The motor holder is connected to the motor link via a flange coupling, while the motor link is further attached to the wrist holder, which is fastened to the user's palm. This exoskeleton allows for movement within a range of 0 to 120°. The design is based on the average dimensions of an adult male hand, measuring 420 mm in length, 100 mm in width, and 80 mm in height. The EULR integrates both mechanical and electronic systems, as shown in Fig. 4, to provide a comprehensive solution for upper-limb rehabilitation.

### 2.2 Hardware circuit

**Fig. 5:**
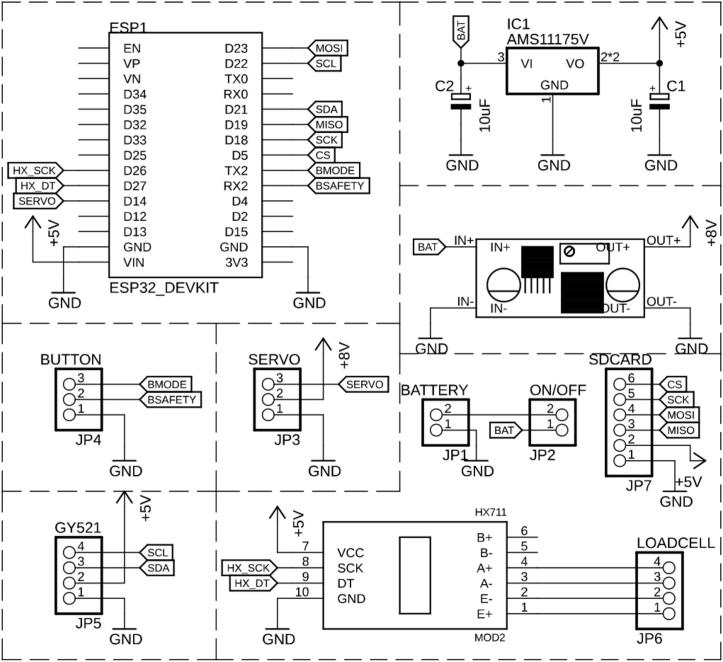
Exoskeleton for Upper Limb Rehabilitation (EULR) circuit.

**Fig. 6:**
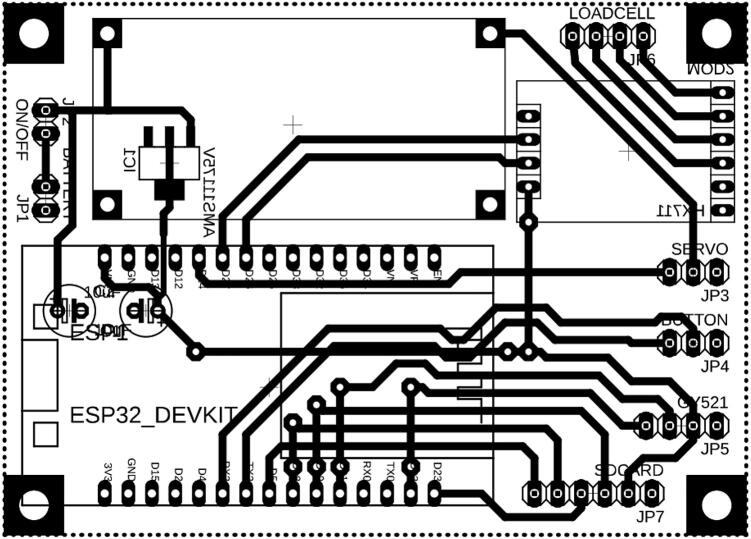
Exoskeleton for Upper Limb Rehabilitation (EULR) printed circuit board.

The hardware circuit is composed of several essential components, with the ESP32 microcontroller acting as the main unit, as shown in Fig. 4. Circuit and printed circuit board design is shown on Fig. 5, Fig. 6, respectively. This microcontroller is powered by the Tensilica Xtensa 32-bit LX6 processor, with its specifications detailed in Table 1. Additional hardware includes five IMU sensors, a load cell sensor, and a servo motor (DS servo, DS5180, China). This servo offers a stall torque of approximately 105 kg·cm at 8 V, with a maximum operating voltage of 8.4 V, and a no-load speed of around 0.19 s per 60°, according to the manufacturer’s datasheet. PWM-controlled servos offer straightforward implementation, precise position control, and high torque output within a compact form factor, making them suitable for wearable rehabilitation devices. Their integrated gearboxes and control electronics simplify system integration and reduce overall system complexity, which is advantageous for rapid prototyping and cost-effective production. The IMU sensor setup utilizes the GY-521 module, which features the MPU6050 integrated circuit. These IMU sensors are strategically placed on the wrist to accurately monitor angular position and movement. The load cell sensor, linked to an HX711 analog-to-digital converter (A/D), delivers precise measurements of force. The A/D output is then sent to the microcontroller through the I2C interface for further processing. This load cell sensor detects arm movements, which trigger the servo motor. The servo motor serves as the main actuator for the exoskeleton arm and is regulated by pulse-width modulation (PWM) signals from the microcontroller, ensuring accurate movement and force application.

**Table 1:** Summary of ESP32 specification.

Items	Specification
MCU	Xtensa Dual Core 32-bit LX6, 600 DMIPS
802.11 b/g/n Wi-Fi	Yes, HT40
Bluetooth	Bluetooth 4.2 and below
Typical Frequency	160 MHz
SRAM	512 kBytes
Flash	SPI Flash up to 16 MBytes
GPIO	36
Hardware/ Software PWM	1/ 16 channels
SPI/ I2C/ I2S/ UART	4/2/2/2
ADC	12-bit
CAN	1
Ethernet MAC Interface	1
Touch Sensor	1
Temperature Sensor	YES
Working Temperature	−40 °C to 12 °C

The power supply circuit is designed around a highly efficient step-down switching regulator, specifically employing the LM2596 module for battery regulation (refer to Fig. 4). This regulator is capable of providing an output current of up to 3.0 A, making it ideal for applications that require substantial power. Central to the system's mechanical functionality is a servo motor, which operates within an input voltage range of 6.0 V–8.4 V. This servo motor can produce a maximum torque of 105 kg.cm and achieves a rotation speed of 0.19 s for every 60°. Furthermore, its operational range extends from 0° to 180°, with movement controlled by pulse-width modulation (PWM) signals that vary between 500 µs and 2500 µs.

**Fig. 7:**
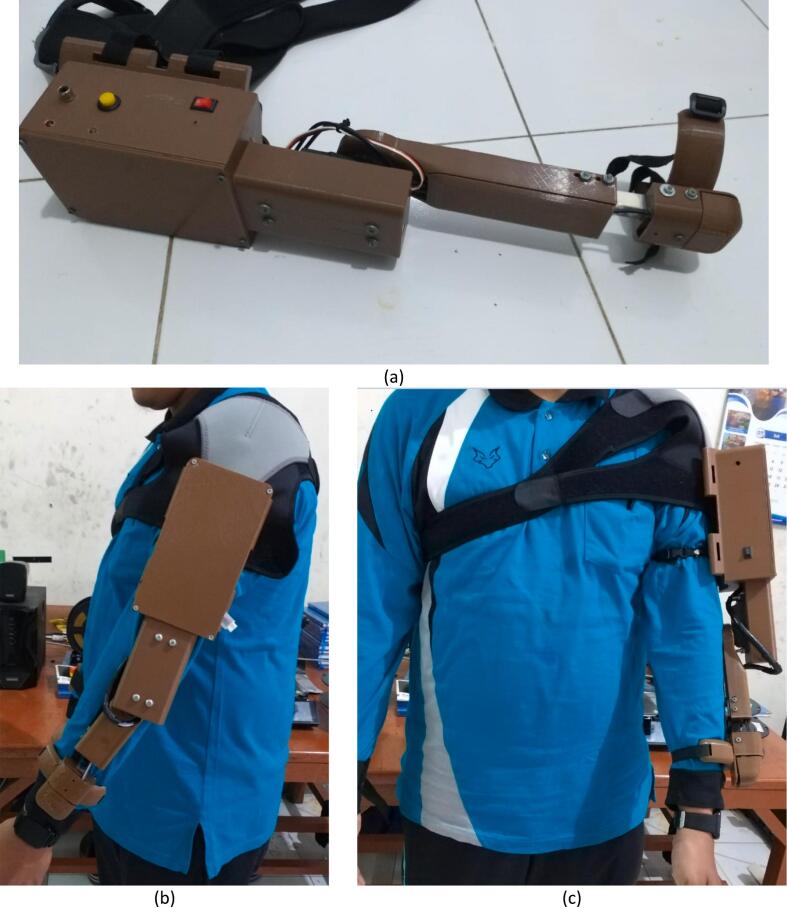
The Exoskeleton for Upper Limb-Elbow Rehabilitation (EULR) design (a) complete design, (b) left view, and (c) front view.

Fig. 7 shows an upper-limb exoskeleton fitted to a respondent, designed to support and assist arm movements. The exoskeleton consists of a harness that secures around the chest and shoulder, providing a stable base for the rest of the apparatus. A rigid frame extends from the shoulder, down the upper arm, and is fastened around the bicep area to stabilize the device and align it with the user’s natural arm structure. At the elbow, there is a joint mechanism allowing flexibility and range of motion, so the device can bend along with the arm while maintaining controlled assistance. The frame continues along the forearm, with an additional strap near the wrist for further stabilization. This configuration suggests that the exoskeleton is intended for mobility assistance or rehabilitation purposes, enabling the user to move their arm more comfortably, potentially with motorized or sensor-based components to support or enhance natural movements.

### 2.3 Exoskeleton for Upper Limb (elbow) Rehabilitation (EULR) firmware

**Fig. 8:**
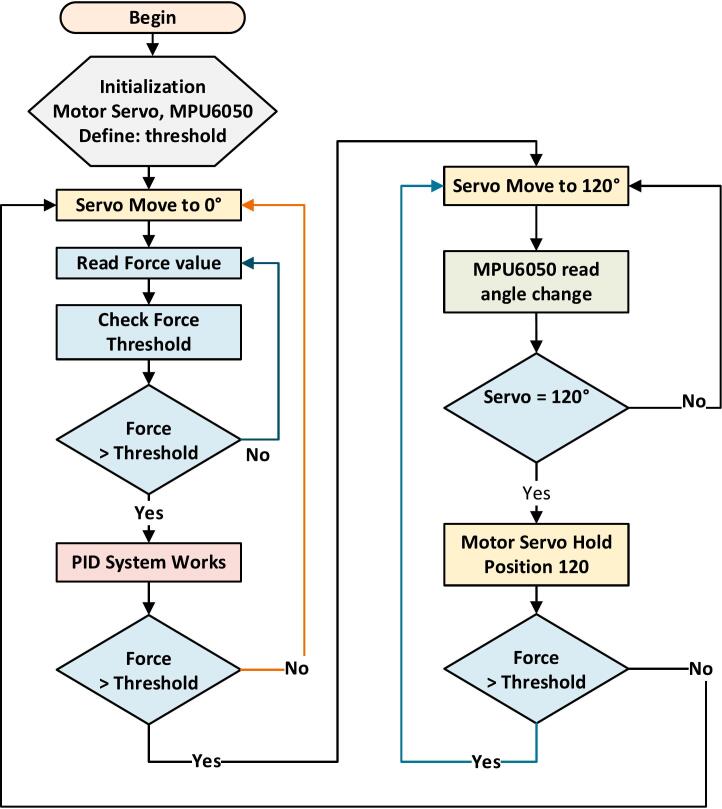
The Exoskeleton for Upper Limb (elbow) Rehabilitation (EULR) flowchart.

The flowchart delineates the control system implemented for the Exoskeleton for Upper Limb Rehabilitation (EULR), which utilizes an Arduino microcontroller (Version 1.8.4, Arduino LLC, New York, US), a servo motor, and an MPU6050 sensor (Fig. 8). This system is engineered to facilitate hand movement by dynamically adjusting the position of the motor in response to feedback from both a force sensor and an angle sensor. The operational sequence commences with the initialization of critical components, specifically the motor servo and the MPU6050 sensor. During this initialization phase, the system establishes a threshold for force measurement, which is pivotal in determining the conditions under which the motor servo should react to external pressures. Subsequently, the servo motor is positioned at an initial angle of 0°. The system continuously monitors force readings to ascertain whether the applied force surpasses the predetermined threshold. If the force remains below this threshold, the motor maintains its position at 0°. Conversely, should the force exceed the threshold, the Proportional-Integral-Derivative (PID) control system is engaged to facilitate precise motor movement. This PID mechanism enables smooth transitions and ensures that the exoskeleton responds in a controlled manner to fluctuations in force. In instances where the applied force continues to exceed the threshold, the servo motor is commanded to shift to a new position of 120°. The MPU6050 sensor is responsible for tracking changes in the motor's angular position. Upon reaching 120°, the system stabilizes at this position. If the force sensor subsequently detects a reduction in force below the threshold, the motor may either remain at 120° or revert to the 0-degree position, contingent upon external conditions. The flowchart effectively illustrates the integration of real-time feedback from sensors and the PID control mechanism, which collectively enable the exoskeleton to adapt to the user's needs. This design is crucial for rehabilitation and daily activities, offering a dependable and responsive system for supporting hand motion.

**Fig. 9:**
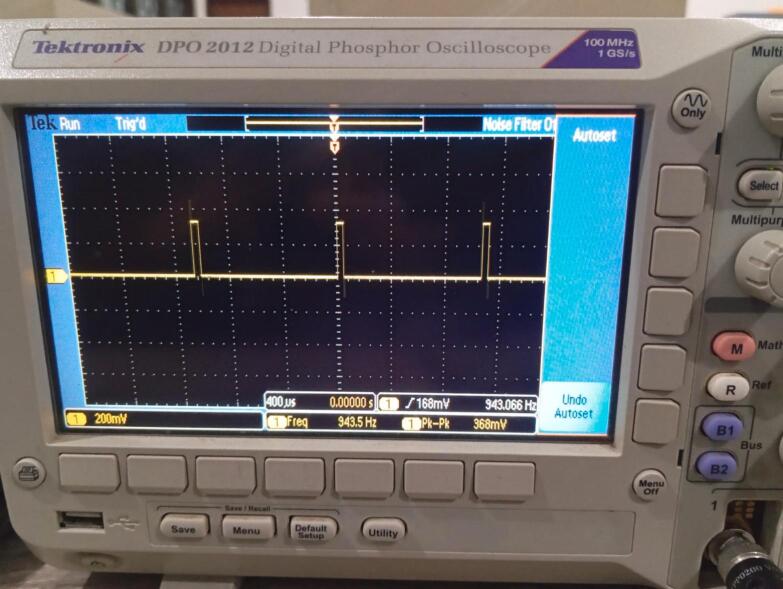
The signal represents the data acquisition activity of the ESP32 microcontroller interfacing with the load cell (via HX711) and the MPU6050 sensor.

In this study, we measured the sampling frequency of the sensor system comprising the 5 kg load cell (with HX711 amplifier) and the MPU6050 inertial measurement unit using a Tektronix DPO 2012 digital oscilloscope (Fig. 9). The sampling frequency was determined by observing the periodic digital signal corresponding to data acquisition events on the communication lines between the ESP32 microcontroller and the sensors. Based on this measurement, the sensor data acquisition rate was approximately 943 Hz. This means that the ESP32 samples and processes sensor data roughly 943 times per second. This high sampling rate ensures sufficient temporal resolution for capturing dynamic changes in force and motion, and is adequate for the control loop responsiveness in our application, particularly in static or quasi-dynamic use cases such as rehabilitation support systems.

### 2.4 Easy to control the EULR

The Exoskeleton for Upper Limb (elbow) Rehabilitation (EULR) is specifically designed to aid in the rehabilitation of post-stroke patients, particularly those who suffer from paralysis in one limb. This innovative device enables individuals to maintain functionality in their unaffected limb while facilitating recovery and movement in the affected arm [19]. The EULR system was developed utilizing 3D printing technology, a microcontroller, high-torque servo motors, a load cell (force sensor), and inertial measurement unit (IMU) sensors. The final product is a lightweight and straightforward open-source exoskeleton for upper limb rehabilitation. A comparative analysis of the weight of this upper limb exoskeleton design relative to findings from other studies is presented in Table 2 [20,21].

**Table 2:** The weight and dimension comparison among other products.

Design	Weight (kg)	Dimension (in)
[20]	4.082	18 × 13 × 6
[21]	3.982	17 × 12 × 6.2
Proposed design	0.98	17 × 11.6 × 6

### 2.5 Cost

**Fig. 10:**
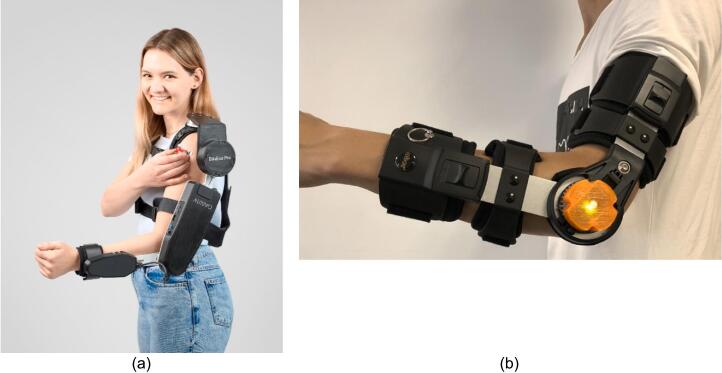
(a) EduExo Pro [20], (b) OREHAB-ARM Smart Brace [21].

The expensive price of commercial exoskeleton products on the market (Fig. 10 (a) US$ 1,990, Fig. 10 (b) US$ > 1.500) as well as the long and expensive rehabilitation process become the obstacles in carrying out post-stroke rehabilitation, especially for people who have middle to low incomes. However, this design has a low cost, namely 98.4 US $ for one EHR product. In addition, this design is open source so that other researchers can develop it with other features and improvement.

### 2.6 Summary

This paper presents the development of a low-cost, 3D-printed hand exoskeleton designed for hand rehabilitation, utilizing force sensor technology. The device aims to enhance accessibility and adaptability for patients, providing a practical solution for rehabilitation therapy.

1. The lightweight exoskeleton is customizable and designed using SketchUp, enabling easy fabrication.
2. It utilizes ESP32 microcontroller and integrates an IMU sensor (MPU6050) and force sensors for accurate monitoring of hand movements.
3. Powered by a rechargeable LiPo battery, ensuring portability and ease of use.
4. The firmware, developed on the Arduino platform, processes sensor data for real-time feedback and controls the servo motors for effective rehabilitation.

## 3 Design files summary

### 3.1 Design file

This section provides a comprehensive overview of the design outputs, encompassing both the hardware and firmware aspects of the upper limb (elbow) exoskeleton system. The hardware design, which includes the schematic diagrams and the printed circuit board (PCB) layout, is detailed in Table 3. Additionally, the firmware developed to control the microcontroller is also outlined in the same table, ensuring seamless operation of the exoskeleton. Moreover, the mechanical design of the hand exoskeleton, which was fabricated using 3D printing technology, is also included as part of this study, offering a complete blueprint for the device's construction and functionality.

**Table 3:** Design file summary of exoskeleton for hand rehabilitation.

Design file name	File type	Open-source license	Location of the file
Circuit	schematic, eagle file	CC BY-SA 4.0	DOI https://doi.org/10.17605/OSF.IO/H8GQK
Arduino Program	firmware, Arduino	CC BY-SA 4.0	DOI https://doi.org/10.17605/OSF.IO/H8GQK
Exoskeleton 3D design	3D printing source, solidworks file	CC BY-SA 4.0	DOI https://doi.org/10.17605/OSF.IO/H8GQK

### 3.2 Schematic and board

The control circuit for the exoskeleton was developed utilizing the Eagle application software (version 6.3.0, free edition for Windows, CadSoft Computer GmbH, Germany). This circuit establishes direct connections to the servo motors, the inertial measurement unit (IMU) sensor (MPU6050), and the load cell sensor, which is interfaced through an HX711 analog-to-digital converter.

### 3.3 Firmware

The firmware for the hand exoskeleton was created using the Arduino application (Version 1.8.4). A detailed overview of the firmware associated with the EULR design is presented in Table 3. This firmware is programmed on the ESP32 microcontroller, which manages all peripheral components, including the system sensors and servo motors. Additionally, the inertial measurement unit (IMU) sensor (MPU6050) is utilized to ascertain the current position of the hand. The microcontroller also processes data from the force sensor. Upon data acquisition, this information is employed to command the servo motor to rotate between 0 and 120°, contingent upon the received input.

### 3.4 3D printing design

The design of the hand exoskeleton was executed using the SolidWorks 2022 software. The 3D design file for the EULR, detailed in Table 3, comprises multiple components, including the servo holder, arm, and wrist holder. Additional information pertinent to the 3D printing design can be found in the referenced materials [22].

## 4 Bill of materials summary

The Bill of Materials (BOM) summary presented in Table 4 provides a comprehensive overview of the components required for constructing the hand exoskeleton, including their quantities, costs per unit, total costs, and sources. The table details essential parts such as the ESP32 microcontroller, high-torque servo motor, load cell force sensor with HX711 interface, MPU6050 IMU sensor, voltage regulators, 3D printing filament, LiPo battery, and connectors. Notably, the total estimated material cost for the device amounts to approximately USD 98.4, effectively demonstrating its low-cost nature. It is important to highlight that the costs listed explicitly cover only the raw materials and components; labor expenses involved in the manufacturing process, such as assembly, calibration, and quality control, are not included in this calculation. This distinction emphasizes the cost-effectiveness of the design from a material standpoint, separate from human labor considerations. Additionally, the presented costs are estimated based on specific regional prices and may differ depending on the purchasing location and prevailing market conditions.

**Table 4:** Bill of materials of a hand exoskeleton.

Designator	Component	Number	Cost per unit (USD)	Total cost (USD)	Source of materials	Material type
Modul microcontroller	Microcontroller ESP32	1	0.99	0.99	Online shop link	semiconductor
Motor	High torque Motor servo 80 kg.cm	1	15.48	15.48	Online shop link	Metal
Force sensor	Loadcell weight sensor 1 kg + HX711	1	0.48	0.48	Online shop link	Steel and semi-conductor
Accelerometer and gyroscope sensor	MPU 6050	1	0.97	0.97	Online shop link	Semiconductor
Adjustable DC supply regulator	LM 2596	2	0.61	1.22	Online shop link	Semiconductor
3D printing materials	PLA Filament 1.75 mm 1 kg Metal Silk PLA 3D Printer Filament	2	7.40	14.8	Online shop link	Polylactic Acid
Battery	11.1 V LiPo Battery	1	4.74	4.74	Online shop link	Lithium phosphat
J1	Female Connectors	1	0.55	0.55	Online shop link	Plastic, metal

## 5 Build instructions

### 5.1 3D printing design

**Fig. 11:**
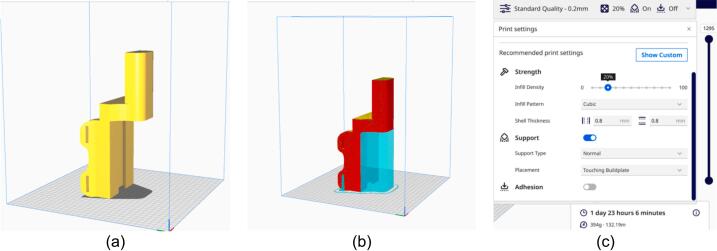
Example of setting to support and build plate adhesion type of raft, (a) object position, (b) supporting position, and (c) print settings.

The hand exoskeleton was fabricated using a 3D printer (Creality CR-10, China) with polylactic acid (PLA) filament. This printer offers precision of 0.015 mm for the X and Y axes, 0.004 mm for the Z axis, and layer precision ranging from 0.1 to 0.4 mm, as specified in the Creality CR-10 user manual [23]. Additionally, the dimensional accuracy of the printed components is maintained at 0.2 mm. The EULR design file, created with SolidWorks (Version 19.0.685 64-bit, 2018 Trimble Inc., Colorado, US), was exported in the STL format, which is a binary file type representing model units. Subsequently, a pre-processing step was performed to convert the STL file into a GCODE file using the Ultimaker Cura application (Version 5.8.1, Ultimaker, Utrecht, Netherlands) (see Fig. 11). The Ultimaker Cura settings were configured for a 30 % infill, resulting in a relatively dense and robust print. It is important to note that higher infill percentages increase both the density of the print and the required printing time, with each section varying in duration based on its volume. Furthermore, the build plate adhesion type was set to “raft” to ensure proper adhesion of the object to the hotbed (refer to Table 5). During the printing process, the nozzle temperature was calibrated to 225 °C, while the bed temperature was set to 60 °C, as indicated in Table 6 for the PLA material (Esun, diameter: 1.75 mm, temperature range: 205–225 °C). The EULR design was tailored to fit an adult (Asian) human size, and the dimensions of the 3D printed components can be adjusted using either the SketchUp application or the settings within Ultimaker Cura.

**Table 5:** Setting parameters on Ultimaker Cura.

Parameter	Unit
Profile	Standard quality (0.2 mm)
Nozzle	0.4 mm
Print speed	80 %
Infill Density	20 %
Infill Line Distance	4.0
Support	Enable
Build plate addesion	Raft extra margin 15 mm

**Table 6:** Setting parameters on 3D printer.

Parameter	Unit
Nozzle temperature	225 °C
Hot bed temperature	60 °C
Print speed	80 %

The current mechanical design was primarily based on average adult male hand anthropometry to ensure functionality for a typical user. While the initial validation focused on this standard size, we recognize the importance of accommodating a diverse range of hand sizes and genders. The STL models used for 3D printing are fully open-source and parametric, allowing for easy customization. Users can modify dimensions using SolidWorks or similar CAD software to tailor the exoskeleton to different hand sizes and anatomies. This flexibility facilitates adaptation across various users, including different genders and age groups.

### 5.2 Firmware design

**Fig. 12:**
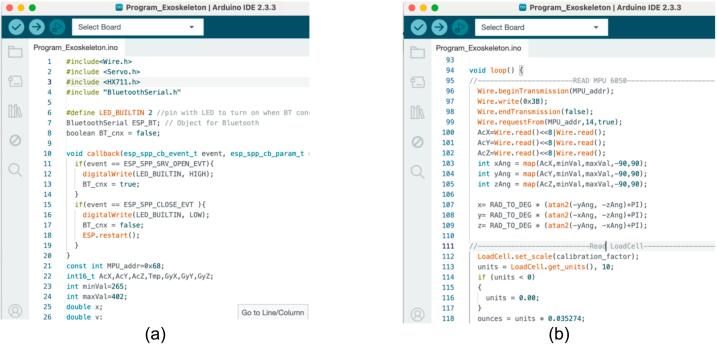
(a) Running Arduino code to obtain the MAC address, (b) placing the MAC address on master program code.

The firmware developed for the hand exoskeleton utilizes the Arduino Integrated Development Environment (IDE) (version 1.8.4) to facilitate real-time control and monitoring of the device's functionalities (Fig. 12). This program is designed to read data from an analog-to-digital converter (ADC) connected to a load cell, enabling the measurement of force exerted by the user. Additionally, it incorporates readings from the MPU6050 sensor, which provides critical information regarding the orientation and motion of the hand. The core of the control system is a Proportional-Integral-Derivative (PID) controller, which processes the input data to ensure precise and smooth adjustments of the servo motor's position. By continuously analyzing the feedback from the load cell and MPU6050, the firmware dynamically drives the servo motor to assist the user in performing rehabilitation exercises, thereby enhancing the effectiveness of the rehabilitation process while ensuring user safety and comfort. This integration of sensors and control algorithms is essential for achieving responsive and adaptive exoskeleton performance.

### 5.3 Hardware circuit

**Fig. 13:**
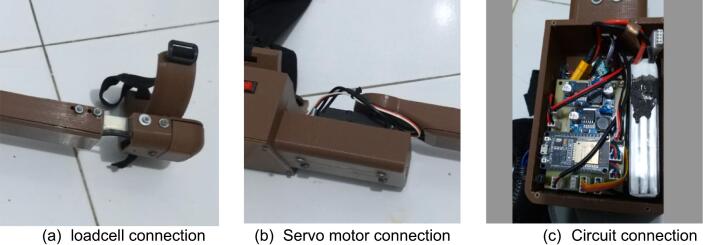
Circuit hardware build instruction.

Once the firmware has been successfully uploaded to the microcontroller, the hardware components are prepared for operational use. The load cell sensor, depicted in Fig. 13(a), is strategically positioned on the wrist to monitor hand movements effectively. This sensor is interfaced with the microcontroller through the HX711 module, which facilitates the conversion of the load cell's analog signals into digital data for processing. Additionally, the Inertial Measurement Unit (IMU) sensor, specifically the MPU6050, is situated at the distal end of the connecting rod. The connectors for the IMU sensors are linked to the ESP32 microcontroller, as illustrated in Fig. 13(a). Servo motors are installed at designated locations on the elbow joint, with a connecting link rod affixed to the wrist to detect flexion and extension movements, as shown in Fig. 13(b). The servo motor arm is securely mounted onto the link rod using four pre-drilled holes, which were incorporated during the 3D design phase, thereby eliminating the need for any additional drilling during assembly. Furthermore, a 11.1 V battery is housed within the circuit box on the circuit board, providing the necessary power for the system. All other peripheral devices are interconnected through the provided cables and connectors, ensuring a cohesive and efficient assembly, as depicted in Fig. 13(c). This systematic arrangement of components not only enhances the functionality of the exoskeleton but also ensures ease of maintenance and adaptability for future modifications.

## 6 Operation instructions

The power supply must be off before using the EULR device. The EULR is placed on the part of the hand that will receive rehabilitation therapy. The EULR design was made for the treatment of a paralyzed left hand. In this case, each of the elastic straps is put on each hand exoskeleton. When the EULR device is ready, the power supply is turned on. Furthermore, the sensor data received by the microcontroller can control the movement of the servo motor connected to the elbow joint. IMU sensor in the wrist part is used to monitor the hand position.

## 7 Validation and characterization

To ensure safety and reliability in human-robot exoskeleton interaction, we conducted mechanical load tests and live movement trials to verify that the servo could handle the expected forces during rehabilitation exercises without reaching stall conditions or causing unintended movements. Additionally, the control system employs real-time feedback from force sensors and position encoders, enabling dynamic adjustments to prevent excessive forces or speeds, thereby enhancing user safety. The calibration of the load cell was conducted under static conditions (Table 7). During this procedure, known static weights were sequentially applied to the load cell using calibrated standard masses, ranging from 50 g to 5000 g (5 kg). At each loading step, the system was allowed to stabilize before the measurement was recorded, ensuring that transient effects were minimized. This approach is appropriate for applications where the sensor is primarily used under static or quasi-static loading conditions. We utilized a portable digital force gauge (Push-Pull Tester Meter, Model SF-200, China) as a reference instrument for calibration. This device has a force resolution of 0.01 N, allowing for sufficient sensitivity across the full calibration range. The linearity error was evaluated by comparing the measured output of the load cell to the actual applied loads. The percentage error at each step was computed, and the mean linearity error of load cell across all data points was 0.2292 %. These results indicate that the load cell maintains good linearity and accuracy within the calibrated range, and is suitable for precise force measurements in static applications.

**Table 7:** Calibration results of the 5 kg load cell using a portable digital force gauge (SF-200, China) under static loading conditions.

Load test(gram)	Calibrator(gram)	Error(%)
50	50.063	0.1260
100	100.564	0.5640
150	150.221	0.1473
200	200.435	0.2175
250	250.407	0.1628
300	300.649	0.2163
350	350.503	0.1437
400	400.115	0.0287
450	450.244	0.0542
500	500.208	0.0416
1000	1007.289	0.7289
1500	1502.486	0.1657
2000	2008.351	0.4175
2500	2504.843	0.1937
3000	3011.914	0.3971
3500	3505.762	0.1646
4000	4010.75	0.2688
4500	4503.561	0.0791
5000	5011.893	0.2379
	Mean error	0.2292

**Fig. 14:**
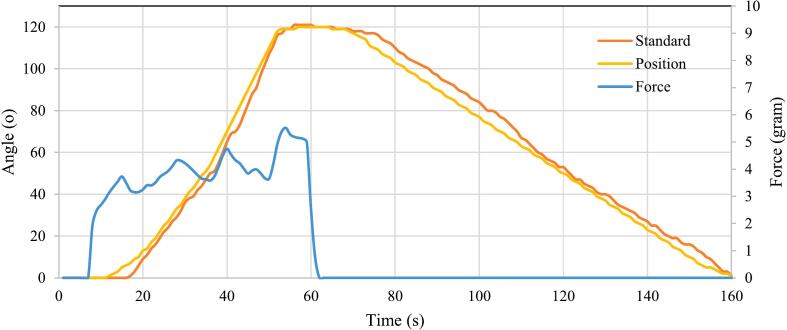
Measurement of the angular position of the hand exoskeleton (flexion and extension) between the sensor IMU (master) and the exoskeleton arm position.

The angular calibration process should be done on the exoskeleton before the validation process. The calibration process was carried out by installing a digital angle measuring instrument (Mini Protractor digital, X15-007, China) on the radius of the hand exoskeleton. Then, this process was performed by adjusting certain angles by positioning the servo motor's rotating angle and measuring the angular position of the resulting hand exoskeleton using a digital angle measuring instrument, as shown in Fig. 14(a). Table 8 shows the results of mean measurements and standard deviations of angles that were performed repeatedly for each setpoint. The mean of standard deviation value obtained for all angular positions measured based on Table 8 is 0.490 ± 0.298 %.

**Table 8:** Measurement of the accuracy of the exoskeleton angle prediction (standard/setpoint is the setting of the position of the exoskeleton arm measured using a digital protactometer measuring device and the prediction is measured using a MPU6050 sensor).

Setpoint (°)	Number of measurements					Mean ± SD.RMSE (°)
	1	2	3	4	5	
0	0	1	0	0	0	1,070 ± 0,282
10	10	10	11	11	12	
30	31	32	30	32	30	
60	62	58	61	60	60	
90	89	89	90	90	90	
120	118	121	121	120	122	

The validation of the hand exoskeleton's motion angles was conducted utilizing an Inertial Measurement Unit (IMU) sensor, which was strategically positioned on the wrist of the exoskeleton, as illustrated in Fig. 14. The validation procedure commenced with the execution of flexion and extension movements of the exoskeleton. Once the hardware system was activated, the elbow joint was capable of performing periodic flexion and extension motions. To ensure that these movements occurred in a consistent and rhythmic manner, the current study employed a metronome application. Specifically, the TempoPerfect Metronome Software (NCH Software, Australia) was utilized to facilitate the timing of the flexion and extension actions, allowing for repetitive and sustained execution of the movements. During the testing phase, the metronome was calibrated to a tempo of 20 beats per minute (bpm), which corresponds to cycles per minute. Fig. 14 provides a visual representation of the angular position data captured by the IMU sensor, comparing the standard movement with that of the exoskeleton.

Regarding the working angle of the motor servo, the DS5180 servo is rated for continuous operation within its specified voltage and load conditions, typically supporting a pulse width modulation (PWM) range between 500 us to 2500 us without thermal issues. During our testing, the servo was operated within these recommended parameters, and no overheating or performance degradation was observed. In terms of temperature rise, we monitored the motor’s temperature during extended operation and found it remained within safe limits, not exceeding 60 °C, which is well below the manufacturer’s recommended maximum operating temperature of approximately 85 °C. Adequate ventilation and voltage management were implemented to prevent excessive heating. For back drivability, the DS5180, like most high-torque servos, exhibits limited back drivability due to internal gear reduction; however, its design allows for controlled resistance aligned with safe interaction. To enhance safety and comfort, the control system incorporates force feedback and compliance algorithms that ensure smooth, responsive movements, preventing abrupt or excessive forces during rehabilitation.

To quantitatively assess the performance of the exoskeleton's hand movements in relation to the glove movements, the root mean square error (RMSE) was calculated, as detailed in Equation (1). This statistical measure serves to evaluate the accuracy of the exoskeleton's motion in replicating the intended movements, thereby providing insights into the effectiveness of the design and control mechanisms employed in the rehabilitation device.

(1) $$\mathrm{RMSE}=\sqrt{\frac{\sum_{i=1}^{N}{\left(y_{i}-x_{i}\right)}^{2}}{N}}$$

where *y_i_* indicates the predicted values, *x_i_* shows the actual values, and *N* is the measurement data. The result of the measurement of the average *RMSE* value for all exoskeleton fingers is 0.498 ± 0.709° (Table 8).

## 8 Conclusion

This study aimed to develop a low-cost, 3D-printed hand exoskeleton that integrates force sensor technology to enhance accessibility and adaptability in hand rehabilitation. The findings demonstrate that the proposed exoskeleton significantly reduces costs to approximately 98.4 USD per unit, making it available alternative to existing commercial devices that often exceed 1,500 USD. Furthermore, the prototype achieved a mean root mean square error (RMSE) of 0.498° ± 0.709° in tracking elbow movements, indicating a high level of accuracy in monitoring hand movements and providing real-time feedback during rehabilitation exercises. Looking ahead, future work will focus on refining the design to improve user comfort and functionality, as well as expanding the range of rehabilitation exercises supported by the exoskeleton. Additionally, further research will explore the integration of advanced machine learning algorithms to enhance the device's responsiveness and adaptability to individual patient needs. Collaborations with healthcare professionals will be sought to conduct clinical trials, assessing the effectiveness of the exoskeleton in real-world rehabilitation settings. By continuing to innovate and improve upon this open-source design, we aim to contribute to the growing field of rehabilitation technology, ultimately enhancing the quality of life for individuals recovering from neuromuscular disorders.

## Ethics statements

The author confirmed that informed consent was obtained from the subjects. This research has passed the ethical examination conducted by Health Research Ethics Committee Poltekkes Kemenkes Surabaya, Indonesia, No. EA/1245/KEPK-Poltekkes_Sby/V/2022.

## CRediT authorship contribution statement

**Triwiyanto Triwiyanto:** Software, Methodology, Conceptualization. **Levana Forra Wakidi:** Project administration, Formal analysis, Data curation. **I. Putu Alit Pawana:** Validation, Supervision.

## Declaration of competing interest

The authors declare that they have no known competing financial interests or personal relationships that could have appeared to influence the work reported in this paper.

## References

[1] Gopura R.A.R.C., Bandara D.S.V., Kiguchi K., Mann G.K.I. (2016). Developments in hardware systems of active upper-limb exoskeleton robots: a review. Rob. Auton. Syst..

[2] Javaid M., Haleem A., Singh R.P., Suman R. (2022). 3D printing applications for healthcare research and development. Global Health J..

[3] Batkuldinova K., Abilgaziyev A., Shehab E., Hazrat Ali M. (2021). The recent development of 3D printing in developing lower-leg exoskeleton: a review. Mater. Today Proc..

[4] Pascual-Leone A. (2013). Robot-assisted therapy in stroke rehabilitation. J Stroke.

[5] Ganguly K., Byl N.N., Abrams G.M. (2013). Neurorehabilitation: motor recovery after stroke as an example. Ann. Neurol..

[6] Chen C.C., He Z.C., Hsueh Y.H. (2011). An EMG feedback control functional electrical stimulation cycling system. J. Signal Process. Syst..

[7] Leonardis D. (2015). An EMG-controlled robotic hand exoskeleton for bilateral rehabilitation. IEEE Trans. Haptics.

[8] Heo P., Member S., Kim J. (2014). Power-assistive finger exoskeleton with a palmar opening at the fingerpad. IEEE Trans. Biomed. Eng..

[9] Ho N.S.K. (2011). 2011 IEEE international conference on rehabilitation robotics.

[10] Jo I., Lee J., Park Y., Bae J. (2017). IEEE International Conference on Rehabilitation Robotics.

[11] Badesa F.J. (2018). Hand exoskeleton for rehabilitation therapies with integrated optical force sensor. Adv. Mech. Eng..

[12] Diez J.A., Blanco A., Catalán J.M., Badesa F.J., Lledó L.D., Garcia-Aracil N. (2018). Hand exoskeleton for rehabilitation therapies with integrated optical force sensor. Adv. Mech. Eng..

[13] A.N.A. Cisnal, S. Member, J. Pérez-turiel, J. Fraile, D. Sierra, E.D.E.L.A. Fuente, RobHand: a hand exoskeleton with real-time EMG-driven embedded control. Quantifying hand gesture recognition delays for bilateral rehabilitation, 2021, pp. 137809–137823. doi: 10.1109/ACCESS.2021.3118281.

[14] Burns M.K., Member S., Pei D., Member S., Vinjamuri R. (2019). Myoelectric control of a soft hand exoskeleton using neural networks and kinematic. IEEE Trans. Biomed. Circuits Syst..

[15] Guo J. (2018). 2018 IEEE International Conference on Soft Robotics (RoboSoft).

[16] Lucas G., Junan C., Minas L. (2018). On the development of adaptive, grasping capabilities enhancement. IEEE Robot. Autom. Lett..

[17] Feys H. (2018). An EMG-controlled robotic hand exoskeleton for bilateral rehabilitation. IEEE Trans. Haptics.

[18] Dwivedi A., Gerez L., Hasan W., Yang C., Liarokapis M. (2019). A soft exoglove equipped with a wearable muscle-machine interface based on forcemyography and electromyography. IEEE Robot. Autom. Lett..

[19] D. Observers, Proof of Concept for Robot-Aided Upper Limb Rehabilitation Using, 45(1) (2015) 110–118.

[20] Gearhart C.J., Varone B., Stella M.H., Busha B.F., Member S. (2016). 2016 38th Annual International Conference of the IEEE Engineering in Medicine and Biology Society (EMBC).

[21] Rose C.G., Malley M.K.O. (2018). A hybrid rigid-soft hand exoskeleton to assist functional dexterity. IEEE Robot. Autom. Lett..

[22] Kukko K. (2020). Additively manufactured parametric universal clip-system: an open source approach for aiding personal exposure measurement in the breathing zone. Appl. Sci. (Switzerland).

[23] Anet 3D, A8 Plus 3D Printer User Manual, 2020. [Online]. Available: https://www.anet3d.com/product/a8-plus-print/.

